# A big data approach to the ultra-fast prediction of DFT-calculated bond energies

**DOI:** 10.1186/1758-2946-5-34

**Published:** 2013-07-12

**Authors:** Xiaohui Qu, Diogo ARS Latino, Joao Aires-de-Sousa

**Affiliations:** 1 CQFB and REQUIMTE, Departamento de Química, Faculdade de Ciências e Tecnologia, Universidade Nova de Lisboa, Caparica 2829-516, Portugal; 2 CCMM, Departamento de Química e Bioquímica, Faculdade de Ciências, Universidade de Lisboa, Campo Grande, Lisbon 1749-016, Portugal

**Keywords:** BDE, Bond dissociation energy, Neural network, Random forest, Machine learning, Chemoinformatics, DFT, DFTB, Big data

## Abstract

**Background:**

The rapid access to intrinsic physicochemical properties of molecules is highly desired for large scale chemical data mining explorations such as mass spectrum prediction in metabolomics, toxicity risk assessment and drug discovery. Large volumes of data are being produced by quantum chemistry calculations, which provide increasing accurate estimations of several properties, e.g. by Density Functional Theory (DFT), but are still too computationally expensive for those large scale uses. This work explores the possibility of using large amounts of data generated by DFT methods for thousands of molecular structures, extracting relevant molecular properties and applying machine learning (ML) algorithms to learn from the data. Once trained, these ML models can be applied to new structures to produce ultra-fast predictions. An approach is presented for homolytic bond dissociation energy (BDE).

**Results:**

Machine learning models were trained with a data set of >12,000 BDEs calculated by B3LYP/6-311++G(d,p)//DFTB. Descriptors were designed to encode atom types and connectivity in the 2D topological environment of the bonds. The best model, an Associative Neural Network (ASNN) based on 85 bond descriptors, was able to predict the BDE of 887 bonds in an independent test set (covering a range of 17.67–202.30 kcal/mol) with RMSD of 5.29 kcal/mol, mean absolute deviation of 3.35 kcal/mol, and *R*^2^ = 0.953. The predictions were compared with semi-empirical PM6 calculations, and were found to be superior for all types of bonds in the data set, except for O-H, N-H, and N-N bonds. The B3LYP/6-311++G(d,p)//DFTB calculations can approach the higher-level calculations B3LYP/6-311++G(3df,2p)//B3LYP/6-31G(d,p) with an RMSD of 3.04 kcal/mol, which is less than the RMSD of ASNN (against both DFT methods). An experimental web service for on-line prediction of BDEs is available at http://joao.airesdesousa.com/bde.

**Conclusion:**

Knowledge could be automatically extracted by machine learning techniques from a data set of calculated BDEs, providing ultra-fast access to accurate estimations of DFT-calculated BDEs. This demonstrates how to extract value from large volumes of data currently being produced by quantum chemistry calculations at an increasing speed mostly without human intervention. In this way, high-level theoretical quantum calculations can be used in large-scale applications that otherwise would not afford the intrinsic computational cost.

## Background

The rapid calculation of physicochemical properties of atoms, bonds and molecules is required to process thousands or millions of structures in data mining explorations, or to establish data-driven sound relationships between structure and observable properties. Quantum chemistry calculations based on *ab initio* and density functional theory (DFT) provide estimations of several properties with increasing accuracy, but with a high computational cost. Many different approximate methods have been developed, such as semi-empirical molecular orbital methods and the self-consistent-charge density-functional tight-binding (SCC-DFTB) method that are 2–3 orders of magnitude faster than DFT and Hartree-Fock using medium-sized basis sets, although with a lower accuracy [1]. Of course, the possibility of applying a specific quantum chemistry method depends on the sizes and numbers of involved molecules, computational resources, as well as time/accuracy requirements.

Density functional theory (DFT) of electronic structure, using approximate exchange-correlation functionals, has enabled the successful application of quantum mechanics to a wide range of problems in chemistry at a fraction of the computational requirements of the traditional Hartree-Fock theory methods [2]. The B3LYP hybrid functional, particularly, is arguably the most popular DFT functional, and has been widely recognized as a cost-effective method. However, DFT calculations are still too computationally expensive (and difficult to automate) for increasingly common data-mining tasks involving thousands/millions of structures, which shall be performed on single workstations or small clusters in time scales of up to a few hours. In fact, even semi-empirical methods are currently hardly affordable in such situations.

At the same time, DFT and *ab initio* calculations are continuously being carried out by many science professionals all over the world producing huge amounts of data. A Big Data scenario can be envisaged in which computational analytic techniques can extract innovative knowledge from the large volumes of data produced by these quantum calculations so that they can be predicted in new situations 5–6 orders of magnitude faster.

Here we present an implementation of this concept in our lab, for the estimation of bond dissociation energies (BDEs). A collection with thousands of molecular structures was retrieved from a public database, and energies were calculated for molecules and fragments by DFT, generating 93.2 GB of data. BDEs were calculated from these data for all non-ring bonds of the compounds, and incorporated into a data set of bonds represented by topological bond descriptors. The data set was used for training machine learning methods, such as Random Forests [3] and Associative Neural Networks, [4] with the goal of establishing models to predict the BDE from the bond descriptors.

BDE is a fundamental thermodynamic property which measures the strength of a chemical bond. It is one of the factors playing a decisive role in the assessment of chemical reactivity, with an impact in different fields, e.g., in determining anti-oxidant activity [5] or identifying the major possible metabolic sites of xenobiotics [6]. The BDE has been used in computational mass spectrometry for the challenging task of identifying unknown compounds in the interpretation of metabolomics data [7]. In order to identify a metabolite, its experimental mass spectrum can be compared to the spectra simulated for the structures of metabolites in large databases, to find the best match. A strategy to identify unknown compounds has consisted in the evaluation of a candidate by generating all its possible topological fragments in order to match the fragment mass with the measured peaks. Candidates are then scored from the number of fragments that explain peaks in the measured spectrum, and the likelihood of the fragmentations has been approached by crude estimations of bond dissociation energies [7].

Some methods have been proposed to predict the BDE, mainly restricted to small data sets of specific types of bonds. Cherkasov et al. [8] developed an additive empirical relationship that was able to predict the homolytic C-H bond dissociation energy within 3.75 kcal/mol for a data set of 79 molecules. The coefficient of determination *R*^2^ was 0.94. However, this relationship is only valid for molecules where resonance contributions and captodative stabilization are insignificant, and the energy range is from 76.4 kcal/mol to 106.7 kcal/mol. Xue et al. [9] developed a Quantitative Structure–property Relationship model of the O-H bond dissociation energy using 78 substituted phenols and Support Vector Machines. A RMSD of 0.79 kcal/mol was achieved for the test set with the dissociation energies ranging from 76.8 kcal/mol to 95.0 kcal/mol. Stanger et al. [10] developed a prediction scheme based on low-level quantum chemistry computation of the hybridization which yielded a correlation coefficient of 0.951 for 35 C-H BDEs. Przybylak et al. [11] developed a relation between the C-H bond dissociation energy of 43 ethers and spin density, which also requires quantum chemistry calculations. Feng et al. [12] constructed a homolytic C-H and N-H bond dissociation energy prediction model for strained hydrocarbons and amines, which is based on quantum chemical descriptors. Two separate models were constructed for hydrocarbon C-H bonds, and amine N-H bonds. Predictions for 89 C-H BDEs were achieved with a correlation coefficient of 0.927 and standard deviation of 2.7 kcal/mol for C-H bonds. The two regression statistical properties are 0.878 and 2.7 kcal/mol for N-H bonds, respectively. Santos et al. [13] studied the homolytic dissociation energies of O-H and S-H bonds in a set of di-substituted phenols and thiophenols by density functional theory at the B3LYP/6-311++G** level. A good agreement between B3LYP/6-311++G** energy and experimental values was observed. Correlations between the bond dissociation energy and Hammet parameters were established in the same work. The correlation coefficient was 0.842 and 0.949 for phenols and thiophenols, respectively.

High-level quantum mechanics theory can be used to directly calculate BDEs. However, as mentioned above, the computational cost is still too high to be used, e.g., in mass spectrum prediction or drug design, which require the ability of on-the-fly evaluation. High-level quantum mechanics calculations require hours to determine the dissociation energy of an individual bond in a small molecule containing 20 or 30 atoms. Unfortunately, the number of atoms of typical drug molecules is far more than 30. A large diverse data set of small molecules and a suitable quantum method are essential for our chemoinformatics data-driven approach, to generate a good quality training set with a large number of bond dissociation energies. The “Fragment-Like” subset of the ZINC database is a practical choice since it includes convenient low molecular weight molecules encompassing a diversity of structural patterns compatible with relevant applications [14,15].

Accurate prediction of bond dissociation energies demands high-level post Hartree-Fork (HF) methods because the electron correlation effects play an important role in the bond breaking process. Coupled Cluster with Single and Double and perturbative Triple excitations (CCSD(T)) and Quadratic Configuration Interaction with Single and Double and perturbative Triple excitations (QCISD(T)) are good examples of such methods. They are able to generate very accurate energies with a sufficient large basis set. However, the choice of a computational method is a balance between accuracy and computational cost. Such methods scale as *N*^7^, where *N* measures the system size, limiting their application to fairly small species for routine use. Density Function Theory (DFT) offers another method to take account of electron correlation effects. DFT methods typically scale as *N*^3^ – *N*^4^ and are able to yield significantly more accurate results than Hartree-Fork (HF) theory. B3LYP is the most popular density functional in chemistry, which has been widely recognized as a cost-effective method and has been successfully applied to a lot of bond dissociation energy research work [2,11-13]. The work here presented employs this method to calculate a database of bond dissociation energies.

Our database involves thousands of species (molecules and fragments). It is possible to calculate the energy for these species using B3LYP. Nevertheless, B3LYP is still too computationally expensive for the geometry optimization and frequency analysis of so many species. The density functional tight-binding (DFTB) is a semi-empirical approximate quantum chemical method derived from DFT by neglect, approximation and parametrization of interaction integrals [1]. The self-consistent-charge density functional tight-binding (SCC-DFTB), which can be derived by a second order expansion of the DFT total energy, extends the DFTB methods to charge self-consistency. The third generation of DFTB methods (DFTB3) was established in 2011 by third order expansion in combination with other improvements, such as the description of coulomb interaction. DFTB3 improves transferability and overall accuracy for several properties. In particular, geometries are usually reproduced excellently [1]. However, because DFTB3 empirical parameter is only available for elements C, H, O, N, S and P, and performance for phosphorous-containing molecules is often unsatisfactory, the current study was restricted to molecules only containing atoms of C, H, O, N, or S.

## Experimental

### Data sets

All the chemical structures in the current study were retrieved from the Fragment-Like database of the ZINC database [14,15]. A subset with the Tanimoto cutoff level of 80% was used, and 1,000 molecules were randomly selected. This data set was randomly partitioned into a training set with 900 molecules (8,336 unique bonds) and a test set with 100 molecules (887 unique bonds). The data sets of molecules were used to build data sets of covalent bonds. In this work, bonds are the objects to be processed by machine learning methods in order to get predictions for their dissociation energies. Additional molecules were retrieved from the Fragment-Like subset to enrich the training set in underrepresented types of bonds (based on atom types of the bond, atom types of neighbors and bond orders). Globally charged molecules and molecules containing elements other than C, H, O, N, or S were discarded. After the enrichment, the training set was screened against itself for identical bonds, which were identified and included only once, finally yielding a training set with 12,834 unique bonds from molecules different from the test set. The 3D structures and BDEs used for training the models are available in the MDL SDFile format in Additional file 1.

### Geometry optimization

The ChemAxon Calculator Plugins were used to generate 3D structures from SMILES strings, and to propose low energy conformers for structure property prediction and calculation, Marvin 5.8.2, 2012, ChemAxon (http://www.chemaxon.com) [16] was employed. For all the molecules in the current study, the lowest energy conformer was used as a starting point for geometry optimization. The geometries of all the species were optimized with the DFTB+ program [17] in combination with the Atomic Simulation Environment (ASE) package [18]. The DFTB+ program was used to calculate the DFTB3 energy and gradient, while the Broyden-Flectcher-Goldfarb-Shanno (BFGS) [19] and the Fast Inertial Relaxation Engine (FIRE) [20] optimizers were used to update the coordinates. The optimized structures were confirmed to be real minima by vibrational frequency analysis (no imaginary frequency). For the radical species with unpaired electrons, spin polarization was enabled in the DFTB calculation.

### Calculation of bond dissociation energies (BDEs) by quantum methods

All DFT calculations were performed using the GAMESS software package [21]. In the current study, the bond dissociation energy is defined as the electronic structure energy change of the following reaction in the vacuum

A−B→A.+B.

in which A and B represent the two fragments formed by breaking the A-B covalent bond in a molecule. The zero point energy (ZPE) correction was not included. After the geometry was optimized for the molecule, the target bond was cut and the molecule was broken into two separate fragments (saved into two SDF files). The geometries of fragments were optimized with DFTB again. Single point energies were calculated for the molecule and for the fragments at B3LYP level in conjunction with the 6-311++G** basis set. Only homolytic dissociation was considered. Therefore, the charge states of both A and B fragments are set to neutral. However, breaking of double bonds originates fragments with two unpaired electron, and both singlet and triplet states are possible. We performed geometry optimization and DFT energy calculation for all possible multiplicities, and used the most stable states to calculate the BDEs.

To assess the reliability of the B3LYP/6-311++G**//DFTB3 energy, bond dissociation energies were also calculated at a higher theoretical level for the whole test set. In those experiments, the geometry optimization was performed at the B3LYP/6-31G** level, and the single point energy was evaluated at the B3LYP/6-311++G(3df, 2p) level.

For a comparison with faster quantum methods, bond dissociation energies were also calculated for the test set with the PM6 semi-empirical method [22]. For this purpose, the MOPAC2009 program was used, [23] the geometry was fully relaxed and confirmed to be real minima by frequency analysis.

Both in the experiments at the B3LYP/6-31G** level and with the PM6 method, some molecules failed to converge in geometry optimization, or had an imaginary frequency, and were not used in the comparisons – 787 bonds were used.

### Bond descriptors

Bond descriptors are required for the processing of bonds by machine learning techniques. Ultra-fast estimation of bond energies cannot rely on computationally expensive descriptors. Therefore, bond descriptors were designed from the outset that: 1) use a simple algorithm; 2) not rely on quantum chemistry calculations; 3) not rely on optimized 3D geometry so that they can be directly calculated from the connection table; 4) not use bond orders explicitly, thus avoiding different mesomers to end up with different descriptors. As the homolytic bond dissociation energy is independent of the bond orientation (A-B or B-A), an additional requirement was that bond descriptors be independent of the bond orientation. With all requirements considered, a set of descriptors was developed to encode a bond (the *target bond*) based on counts of atom types and pairs of atom types existing in the bond or in its neighborhood.

The software for the calculation of bond descriptors was written in the Java programming language, and relies on the Chemistry Development Kit (CDK) libraries [24]. Therefore, in this implementation, definitions of aromaticity and π-systems are provided by the CDK *CDKHueckelAromaticityDetector* algorithm. The source code to calculate bond descriptors is available at http://sourceforge.net/projects/unlpredict.

#### Atom type

Two kinds of atom types are used in this work. The first simply consists of classifying an atom according to its element (C, H, O, N, S). The second definition of atom type combines the element and the number of connected atoms. For example, the carbon atom in methane is C4, the carbon atom in ethylene is C3 and the carbon atom in acetylene is C2. There are 14 types defined in the current study: C2, C3, C4, H1, N1, N2, N3, N4, O1, O2, S1, S2, S3, S4. Hereafter, we will use the term “connection number atom type” to refer to this latter atom type system.

#### Spheres

**Figure 1:**
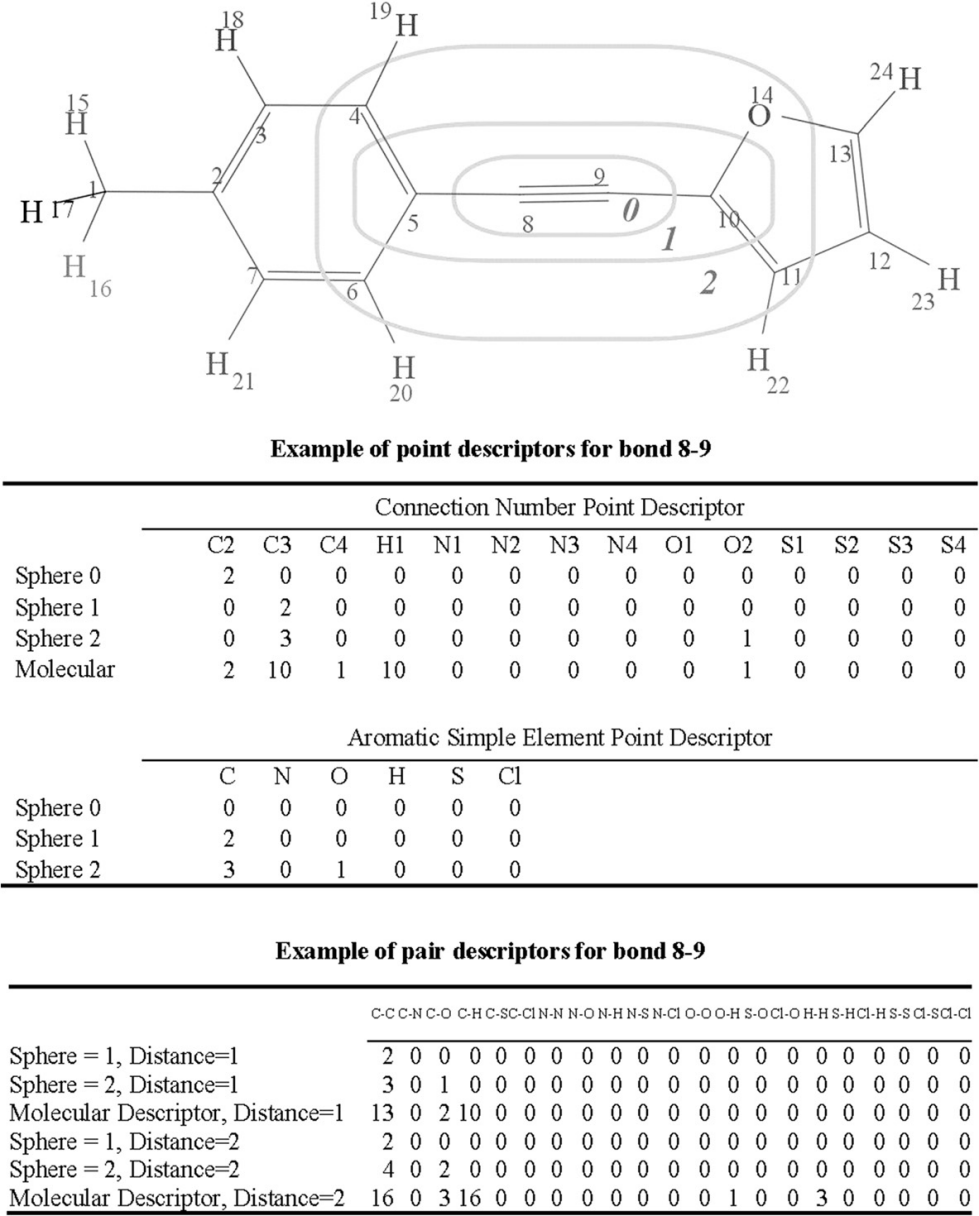
Examples of bond descriptors.

To encode the distance between the target bond and atoms in the molecule, descriptors are computed at different layers (*spheres*). The classification of atoms into different spheres is based on the distance to the target bond (number of covalent bonds between the atom and one atom of the target bond on the shortest possible path). As shown in Figure 1, atoms 8 and 9 are involved in the target bond and belong to sphere 0; atoms 5 and 10 are one bond away from bond 8–9 and belong to sphere 1; sphere 2 includes the atoms 4, 6, 11, and 14. Initially, up to 7 spheres (from 0 to 6) were taken into account.

#### Point descriptors

These are counts of specific atom types in a specific sphere. If the number of atom types is *N*, the point descriptors for a single sphere is an array of size *N*. This descriptor can be calculated for the different systems of atom types. *Element point descriptors* correspond to point descriptors based on element atom types; *connection number point descriptors* correspond to point descriptors based on connection number atom types. The descriptors can be restricted to subsets of the molecule. For example, restriction to aromatic atoms will result in aromatic point descriptors. Figure 1 shows several examples of bond descriptors.

#### Pair descriptors

These are counts of atom pairs of specific atom types in specific spheres at specific distances between them. Pair descriptors for a sphere are defined so that one atom of the pair belongs to the sphere and the other atom is in the same or in a lower sphere. Pair descriptors are specified for a distance, which is the number of bonds between the two atoms of the pair on the shortest possible path. Figure 1 illustrates element pair descriptors. For example, for bond 8–9, the pair descriptor for sphere 2, distance 2, atom types “C-C” is 4, which results from counting atom pairs 4–6, 4–8, 6–8 and 11–9. In this paper, we used pair descriptors with different combinations of spheres, distances and atom types.

Pair descriptors can also be calculated without atom types, i.e., counting all the pairs of specific spheres and distances regardless of the atom types – *no-type pair descriptors*. The main advantage of no-type pair descriptors is low dimensionality; when compared with a large atom type system, this reduction is particularly relevant.

#### Bond-breaking difference pair descriptors

To describe the bond breaking explicitly, and at the same time obtain descriptors independent of the bond orientation, descriptors were also defined as the difference between pair descriptors before and after breaking the target bond – *bond-breaking difference pair descriptors.* Breaking the bond generates two fragments, and pair descriptors after the cleavage derive from atom pairs residing on the same fragment.

#### Molecular descriptors

The number of atoms or pairs can also be counted in the whole molecule without restriction to spheres of a target bond. These are *molecular descriptors*, which are the same for all the bonds in the same molecule. Examples of molecular point descriptors and molecular pair descriptors are shown in Figure 1.

#### Fragment point descriptors

These are point descriptors calculated for individual fragments after cleavage of the target bond. For each specific point descriptor (defined for a sphere and atom type) there are two values, one for each fragment. In order to be independent from the bond orientation, the two values are sorted. The main purpose of this descriptor was to provide information on the distribution of special functional structures, such as aromatic systems, or conjugated π systems, by restricting the atoms involved in the calculation of the descriptors.

### Machine learning methods

The relationship between bond descriptors and DFT-calculated bond dissociation energies was explored with two different machine learning algorithms, Random Forests (RF) [3] and Associative Neural Networks (ASNN) [4].

Random Forests (RF) were employed as ensembles of unpruned regression trees created by using bootstrap samples of the training data. The best split at each node is defined among a randomly selected subset of descriptors. Prediction is made by an average of the individual regression trees in the forest. This is a high-dimensional nonparametric method that works well on large numbers of variables. The performance is internally assessed with the prediction error for the objects left out in the bootstrap procedure (out-of-bag estimation, OOB). Here, RFs were grown with the R program version 2.14.1, [25] using the randomForest library [26]. The number of trees in the forest was set to 1,000, and the number of variables tested for each split was set to default (square root of the number of variables).

Associative Neural Networks (ASNNs) integrate an ensemble of Feed-Forward Neural Networks (FFNNs) with a memory of data. The ensemble consists of independently trained FFNNs, which contribute to a single prediction. The ASNN scheme is employed for composing a prediction of the bond dissociation energy from a) the outputs produced by the ensemble of NNs and b) the most similar cases in the memory (here, the training set). The ASNN program was kindly provided by Dr. Igor Tetko. It was used with the Levenberg-Marquardt algorithm to train fully connected FFNNs with an input layer (including a bias equal to 1), one hidden layer (also including a bias equal to 1), and one output neuron. The presence of a bias enables to shift the activation function upwards or downwards by an adjustable value. The number of hidden neurons was optimized based on the training set, and in the final experiments was set to 6. The logistic activation function was used and each input and output variable was linearly normalized between 0.1 and 0.9 on the basis of the training set. Prior to the training of each network, the program randomly divided the training set into a validation set and a reduced training set with approximately the same size. Full cross-validation of the training set was performed using the leave-one-out method (LOO). The training was stopped when there was no further improvement in the root mean square deviation (RMSD) for the validation set.

## Results and discussion

### Random forest prediction of bond dissociation energies

In a first experiment, random forests were trained with an extensive pool of 3,675 bond descriptors, to predict the bond dissociation energies calculated with B3LYP/6-311++G(d,p)//DFTB. All the descriptors were based on the connection number atom type system, and consisted in 1) point descriptors (including molecular point descriptors), 2) point descriptors of atoms in ring systems, 3) point descriptors of atoms in π systems, 4) point descriptors of atoms in aromatic systems, 5) pair descriptors, 6) bond-breaking difference pair descriptors. All the descriptors were calculated for 7 spheres (the maximum sphere radius is 6 bonds). In pair descriptors, interatomic distances were considered from 1 to 4 bonds. Bonds with identical descriptors were detected and included only once in the training set.

A random forest with 1,000 trees was able to predict the B3LYP bond dissociation energy with Mean Absolute Deviation (MAD) of 4.24 kcal/mol, and Root Mean Square Deviation (RMSD) of 6.87 kcal/mol. These results were obtained with the out-of-bag (OOB) validation on the training set. The bond dissociation energies in the training set cover a range between −9.19 kcal/mol and 226.88 kcal/mol. The minimum positive bond dissociation energy is 0.45 kcal/mol. Only 14 bonds obtained negative values, which are probably the result of numerical errors of the B3LYP energy calculation, and in this experiment they were included in the training set. All the 14 bonds are weak, and ten of them are N-N_2_ bonds of azides. A non-dynamical electronic correlation effect is likely to be important – but DFT is a single configuration quantum chemical method that only takes dynamical electronic correlation into account. Another possible reason is the insufficient description of the non-covalent interactions of B3LYP, such as hydrogen bonds, van der Waals interactions, or charge-transfer interactions. Four of the 14 structures contain simultaneously atoms with formal positive charge and atoms with formal negative charges, in which non-covalent interactions are thus expected to occur. On the other hand, it has been noted that B3LYP systematically underestimates BDEs [27] – the negative BDEs can be viewed as a particular case of underestimation.

This first model employs a very large number of descriptors. Although the encoding of all the relevant information requires a sufficiently detailed representation of the bond atoms and neighborhood, it was expected that a selection of descriptors could be found that would enable at least the same quality of the predictions. A series of experiments were carried out with that goal. Replacement of descriptors based on connection number atom types by simple element atom types reduced the number of descriptors from 3675 to 615 (Selection 1). However, the accuracy of RF predictions also deteriorated significantly. The MAD increased by 27%, while the RMSD increased by 30%, indicating that the more sophisticated atom type system is vital for the success of bond dissociation energy prediction. Then we tried to use the connection number atom types for point descriptors (the number of descriptors scales as O(*N*)), the simple element atom types for pair descriptors of distance 1, and no-type pair descriptors of larger distances (2–7 bonds) (Selection 2). The number of pair descriptors scales as O(*N*^2^). To some extent, the aromatic and the π-system point descriptors overlap with the general point descriptors, and were replaced by fragment point descriptors. Table 1 shows the performance of the two selection strategies compared to the original model with all the descriptors. The second strategy was much more successful. It could reduce the number of descriptors to 293 while maintaining essentially the same prediction accuracy (just a 2% increase for both MAD and RMSD). Encouragingly, the maximum error decreased by 14.71 kcal/mol, while it increased by 10.95 kcal/mol with the first approach. Table 1 also shows some decrease in the model performance when only connection number point descriptors were used.

**Table 1:** **Random Forest prediction of bond dissociation energies with different selections of descriptors obtained in the out-of-bag (OOB) validation procedure over the training set **^**a**^

**Atom types**	**No. of descriptors**	**RMSD**	**MAD**	**MaxError**
Connection Number	3675	6.87	4.25	71.58
Connection Number (only point descriptors)	112	7.50	4.77	79.55
Simple Element (Selection 1)	615	8.90	5.41	82.53
Selection of connection number, element, and no-type (Selection 2)	293	7.01	4.35	56.87

^a^ Results are in kcal/mol.

To test the importance of the various groups of descriptors, we retrained the RF after selectively removing one group of descriptors from Selection 2, and this was performed for each of 10 groups of descriptors (Table 2). Clearly, the point descriptors based on connection number atom types are more important than the others, which is understandable since they provide a detailed information on the availability of specific types of atom in the bond and in the spheres around the bond. Based on the relative importance of the groups of descriptors, and also on the number of descriptors in each group, a new selection was put forward (Selection 3) with connection number atom types point descriptors, element pair descriptors, aromatic fragment point descriptors (with the corresponding molecular descriptors), no-type pair descriptors, and π system fragment point descriptors (with the corresponding molecular descriptors). The new selection consisted of 209 descriptors, and enabled to train an RF yielding essentially the same prediction accuracy (Table 2). The absence of bond-breaking difference pair descriptors in this selection suggests that the relevant information they would possibly encode is included in the patterns generated by other groups of descriptors. It was found that the π system fragment point descriptors could be merged for the two fragments (summing their values - *π total number descriptors*) without changing results (201 descriptors).

**Table 2:** **Impact of individual groups of descriptors on random forest prediction of bond dissociation energies **^**a**^

	**No. of descriptors**	**RMSD**	**MAD**	**MaxError**
Selection 2 ^b^	293	7.01	4.35	56.87
Selection 2 - CN point	209	9.06	5.50	80.97
Selection 2 - Element pair	218	7.18	4.48	57.13
Selection 2 - Fragment point	288	7.02	4.36	58.75
Selection 2 - Aromatic fragment point	279	7.07	4.39	62.14
Selection 2 - In-ring fragment point	281	7.03	4.36	55.31
Selection 2 - No-type pair	269	7.06	4.43	58.95
Selection 2 - No-type bond-breaking difference pair	284	6.96	4.32	57.70
Selection 2 - π fragment point	281	7.03	4.37	58.59
Selection 2 - Molecular element pair	263	6.96	4.31	57.86
Selection 2 - Molecular CN fragment point	265	7.04	4.35	57.77
Selection 3 ^c^	209	7.00	4.32	58.36

^a^ Results are in kcal/mol, and were obtained in the out-of-Bag (OOB) RF validation procedure over the training set.

^b^ Combination of the following descriptors: 1) Connection number point descriptors, 2) Simple element pair descriptors, 3) Fragment point descriptors (only the field with the lower value is used), 4) Aromatic fragment point descriptors with corresponding molecular descriptors, 5) In-ring fragment point descriptors, 6) No-type pair descriptors, 7) No-type bond-breaking difference pair descriptors, 8) Conjugated π system fragment point descriptors with corresponding molecular descriptors, 9) Simple element molecular pair descriptors, 10) Molecular connection number fragment point descriptors. Descriptors 1, 4, 5, are calculated for spheres from 0 to 5. Descriptors 2, 3, 6, 8 are calculated for spheres from 1 to 5. Descriptor 2 only involves pairs at a distance of one bond. Descriptor 7 involves pairs with interatomic distances from 2 to 7. Descriptor 9 involves pairs with interatomic distances of 1–2.

^c^ Combination of Descriptors 1, 2, 4, 6, 8.

Another important factor regarding the size of the descriptor set is the number of spheres used. Table 3 shows results for different number of spheres. It can be concluded that reducing the maximum number of spheres to 4 does not significantly affect the quality of predictions, but further reduces the number of descriptors considerably – it is a satisfying balance of accuracy and model size, and is kept for further studies. Finally, the 90 most important descriptors reported by this last RF were selected according to the Mean Decrease Accuracy (%InMSE), and were used to build the final random forest model. Table 4 shows the number of selected descriptors from each group.

**Table 3:** Random forest prediction of BDEs with descriptors encoding different spheres around the target bond

**Nr of spheres**	**No. of descriptors**	**RMSD**	**MAD**	**MaxError**
1	31	11.97	7.91	121.82
2	60	7.95	5.22	56.00
3	94	7.21	4.59	55.62
4	129	7.10	4.46	59.41
5	165	7.04	4.38	60.22
6	201	7.00	4.33	57.67
7	240	7.01	4.32	57.33
8	278	7.01	4.34	57.59
9	316	7.03	4.35	57.41
10	354	7.04	4.35	57.20

**Table 4:** Descriptors selected by random forest to predict bond dissociation energies

**Group of descriptors**	**Available**	**Selected**
CN point desc.	56	39
Element pair desc.	45	23
Aromatic fragment point desc.	10	10
No-type pair desc.	15	15
π Total number desc.	3	3

Out-of-bag validation yielded RMSD of 7.09 kcal/mol, MAD of 4.46 kcal/mol, *R*^2^ = 0.952 and the maximum error was 56.29 kcal/mol. At the end, the model was tested with the independent test set consisting of bonds from 100 molecules. The RMSD was 6.04 kcal/mol, the MAD was 3.84 kcal/mol, *R*^2^ = 0.939 and the maximum error was 37.07 kcal/mol. The better results for the test set, comparing to the training set, may be due to the high diversity of the training set, which was designed to include reasonable numbers of underrepresented, hard-to-predict types of bonds.

### Prediction of bond dissociation energies with Associative Neural Networks

**Figure 2:**
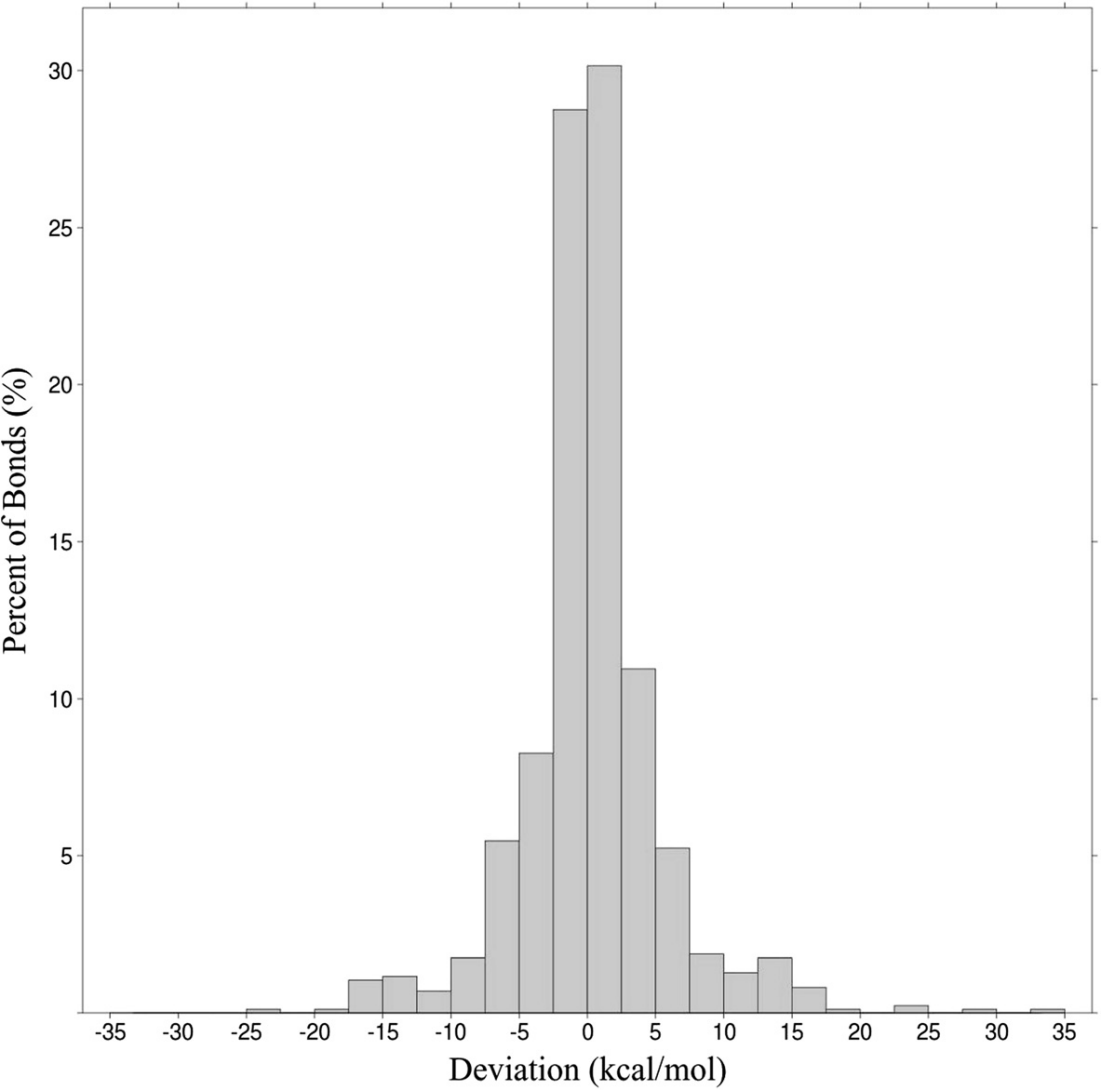
Distribution of ASNN BDEs errors in the test set.

**Figure 3:**
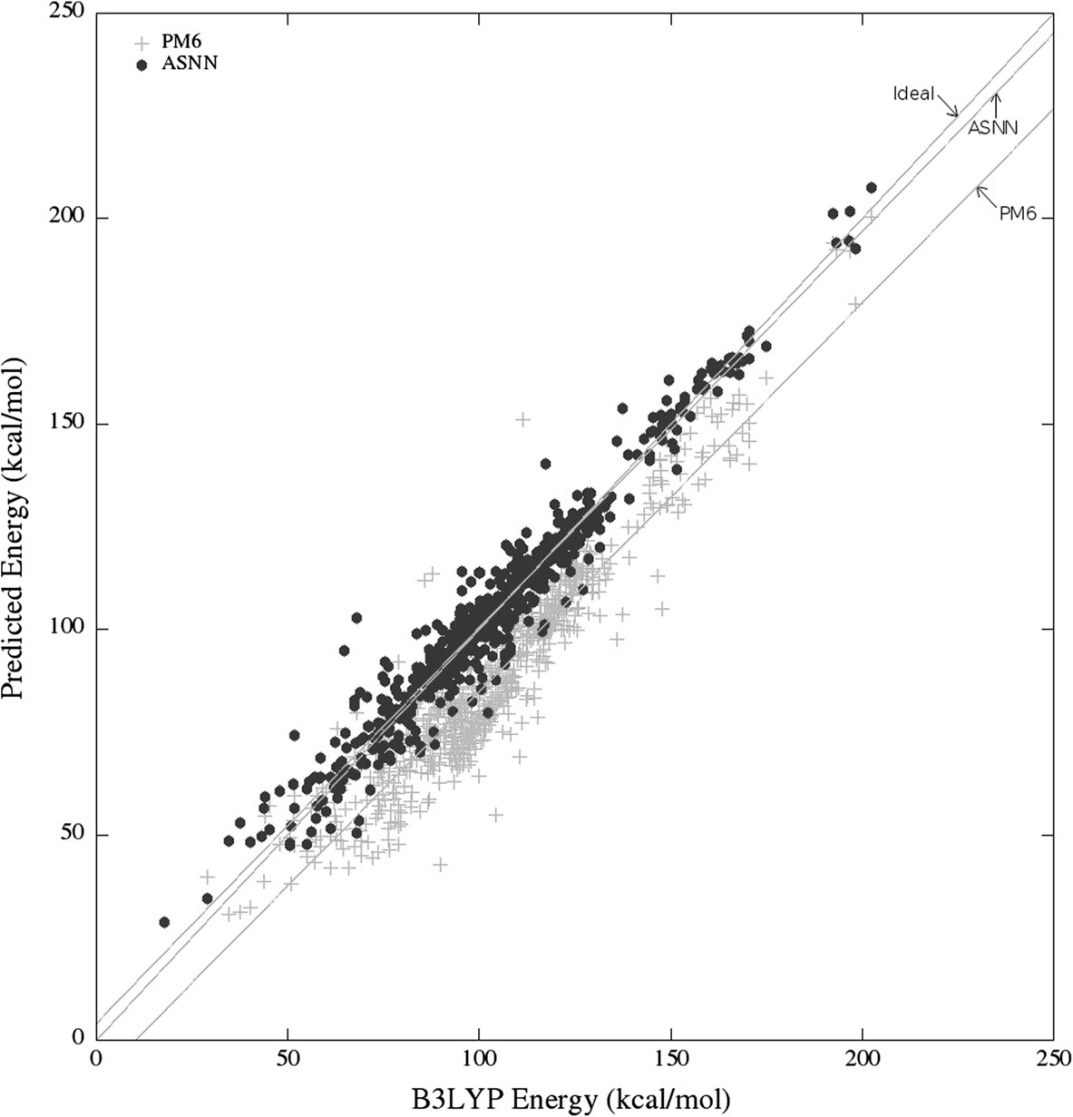
ASNN and PM6 predictions of BDEs versus B3LYP calculations.

The results could be further improved by training an Associative Neural Network (ASNN) model with the 90 descriptors previously selected (five descriptors were further removed due to high correlation to other descriptors). The number of hidden neurons was set at 6, and 125 networks were used in the ensemble. The ASNN could predict the bond energies of the independent test with RMSD 5.29 kcal/mol, MAD 3.35 kcal/mol, and *R*^2^ 0.953. Figure 2 illustrates the distribution of errors in the test set. In terms of computation time, the whole procedure for the 100 molecules of the test set, starting from the MDL SDFiles takes less than 5 s in a contemporary PC (Intel Core i7-870 2.93 GHz, 8 GB RAM). The accuracy of the predictions obtained by this ultra-fast model were compared with calculations obtained by the semi-empirical PM6 method. The results are presented in Table 5 and Figure 3. While the PM6 shows a systematic deviation from the B3LYP energy, the ASNN model reproduces the bond dissociation energy accurately, and is superior even by comparing *R*^2^ values. The accuracy of the predictions is well maintained for different types of bonds (Table 5), with the exception of O-H, N-H and N-N bonds. For these, the PM6 method achieved better predictions according to the *R*^2^ parameter (and for the O-H bonds also according to RMSD). A reason for the difficulties in predicting O-H and N-H bonds can be hydrogen bonds. These can significantly influence bond energies, but depend on 3D geometries and through-space interactions between atoms further apart than our bond descriptors can account for. In a big data scenario, ASNNs have the interesting possibility of gradually incorporating more data in its memory, for more accurate predictions, without retraining the networks.

**Table 5:** **Accuracy of dissociation energies predicted by ASNN, RF and calculated by PM6 (against B3LYP-calculated BDEs) for different types of bonds **^**a,b**^

	**PM6**				**RF**				**ASNN**			
	**RMSD**	**Max Error**	**MAD**	***R***^***2***^	**RMSD**	**Max error**	**MAD**	***R***^**2**^	**RMSD**	**Max error**	**MAD**	***R***^**2**^
C-C	21.38	49.57	19.27	0.806	6.64	23.83	4.69	0.907	5.47	22.95	3.72	0.938
C-H	18.06	35.48	17.15	0.869	3.77	19.79	2.34	0.942	3.68	22.64	2.20	0.944
C-N	17.30	39.60	15.10	0.919	8.52	26.15	5.87	0.928	6.70	17.33	5.02	0.955
C-O	13.97	24.54	12.45	0.969	6.73	21.10	4.96	0.971	5.42	16.67	3.87	0.979
C-S	7.78	14.83	6.42	0.881	5.99	14.30	4.53	0.887	4.29	10.46	3.20	0.950
N-H	12.04	20.45	11.03	0.799	8.39	37.07	5.47	0.576	8.31	34.99	5.19	0.586
O-H	10.73	15.45	9.58	0.975	12.35	24.78	9.02	0.704	12.46	22.54	9.53	0.586
N-N	13.06	22.99	10.21	0.779	13.03	21.95	12.14	0.514	10.10	15.12	8.47	0.736
N-O	10.51	18.85	8.72	0.635	5.01	9.11	3.89	0.912	7.58	17.63	5.07	0.827
S-O	25.90	26.04	25.90	1.000	0.69	0.78	0.69	1.000	1.94	2.01	1.94	1.000

^a^ 787 bonds were used for the comparison, from the molecules of the test set that could be calculated with PM6.

^b^ Each line corresponds to a type of bond between atoms of specific elements (all bond orders included).

For simple applications, typical energies for bonds of specific orders between atoms of specific elements are often used as “fixed” predictions. This possibility was evaluated by calculating the average bond energy in the training set for bonds of specific orders between atoms of specific elements, and using these “fixed” values as predictions for the bonds of the same type in the test set – Table 6. For example, the average of BDEs for carbon-nitrogen single bonds in the training set is 82.95 kcal/mol, therefore the “fixed” prediction for all carbon-nitrogen single bonds in the test set is 82.95 kcal/mol. Table 6 confirms that the quality of the ASNN model is far superior to that of the fixed values.

**Table 6:** **Comparison between BDEs predicted by ASNN and BDEs predicted as the average of BDEs for the bonds of the same type in the training set **^**a**^

	**Fixed values**			**ASNN**			
**Bonds**	**RMSD**	**MaxError**	**MAD**	**RMSD**	**MaxError**	**MAD**	***R***^**2**^
C-C	21.64	62.88	16.56	5.47	22.95	3.72	0.938
C-H	15.48	58.64	12.94	3.68	22.64	2.20	0.944
C-N	20.57	65.28	15.65	6.70	17.33	5.02	0.955
C-O	14.18	46.63	10.87	5.42	16.67	3.87	0.979
C-S	11.03	23.36	8.85	4.29	10.46	3.20	0.950
H-N	13.20	33.17	10.17	8.31	34.99	5.19	0.586
H-O	22.65	44.56	15.62	12.46	22.54	9.53	0.586
N-N	21.81	42.80	18.12	10.10	15.12	8.47	0.736
N-O	8.65	15.21	7.44	7.58	17.63	5.07	0.827
O-S	1.85	2.55	1.58	1.94	2.01	1.94	1.000

^a^ Each line corresponds to a type of bond between atoms of specific elements and of a specific order.

To assess the reliability of the B3LYP/6-311++G**//DFTB3 energy, bond dissociation energies were also calculated at a higher theoretical level for the whole test set (geometry optimized at the B3LYP/6-31G** level, and the single point energy evaluated at the B3LYP/6-311++G(3df, 2p) level). Correlations between these results, the DFTB3 calculations, the PM6 calculations, and the ASNN predictions are displayed in Table 7, represented as RMSD, MAD, and *R*^2^. They show that the DFTB3 calculations approach the higher-level calculations with an RMSD of 3.04 kcal/mol, which is less than the RMSD of ASNN (against both methods). It indicates that training of ASNN with more accurate data would probably not improve the performance of the ASNN. It also demonstrates how ultra-fast data-driven chemoinformatics methods can be competitive with semi-empirical methods.

**Table 7:** **Correlations between BDEs calculated at different levels of theory and estimations by PM6 and ASNN **^**a**^

	**B3LYP/6-311++G(3df,2p)//B3LYP/6-31G(d,p)**				**B3LYP/6-311++G(d,p)//DFTB**			
	**RMSD**	**MAD**	**MaxError**	***R***^**2**^	**RMSD**	**MAD**	**MaxError**	***R***^**2**^
B3LYP/6-311++G(d,p)//DFTB	3.04	1.82	21.41	0.985				
PM6	16.88	15.46	46.03	0.901	17.52	15.98	49.57	0.890
ASNN	5.18	3.38	33.58	0.956	5.16	3.21	34.99	0.954

^a^ 787 bonds were used for the comparison, from the molecules of the test set that could be calculated with all DFT and PM6 methods.

A web service incorporating the new ultra-fast methods has been made available for online estimation of BDEs - http://joao.airesdesousa.com/bde. For operational reasons, it was more convenient to implement the web service based on an ensemble of FFNNs with the Weka package [28]. A bootstrap aggregating ensemble (bagging) was formed with 75 neural networks containing 6 hidden neurons each. It was further improved by additive regression which consists of 5 iterations (bagging ensembles). It was confirmed that the quality of the predictions was essentially the same as with the ASNN.

### Limitations of the current model

In order to build a fast data-driven method, the representation of molecular structures must be simplified. The main simplification was the definition of descriptors from the connection table regardless of any 3D structure. *Trans* and *cis* isomerism is also not accounted for. Furthermore, during the selection of descriptors, the maximum sphere around the target bond was reduced to 3. This occurred probably because most of the factors determining bond energies (up to a certain level of accuracy) are within 3 bonds of the target bond. However, such a reduction precludes the model from distinguishing bonds whose differences are only outside of the 3rd sphere. The following two examples illustrate some of these limitations.

**Figure 4:**
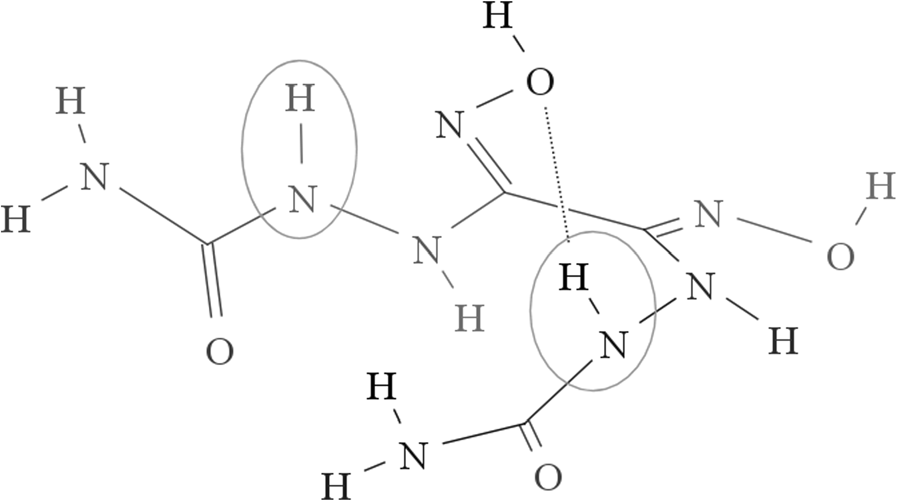
Example of a symmetrical molecule with different BDEs for two topologically equivalent bonds due to hydrogen bonding.

The molecule displayed in Figure 4 is symmetrical and includes two topologically equivalent N-H bonds. However, in the lowest energy conformer, one of the two is involved in a hydrogen bond with the hydroxyl group, which leads to an extra energy cost for bond dissociation. As a result, the dissociation energies of the two N-H bonds differ by 10.29 kcal/mol. Because the calculation of descriptors depends solely on connectivity, the descriptors are the same for the two bonds, and there is no way for our model to predict any difference in the bond dissociation energy. This means the current model cannot be expected to predict bond dissociation energies that are strongly conformation-dependent.

**Figure 5:**
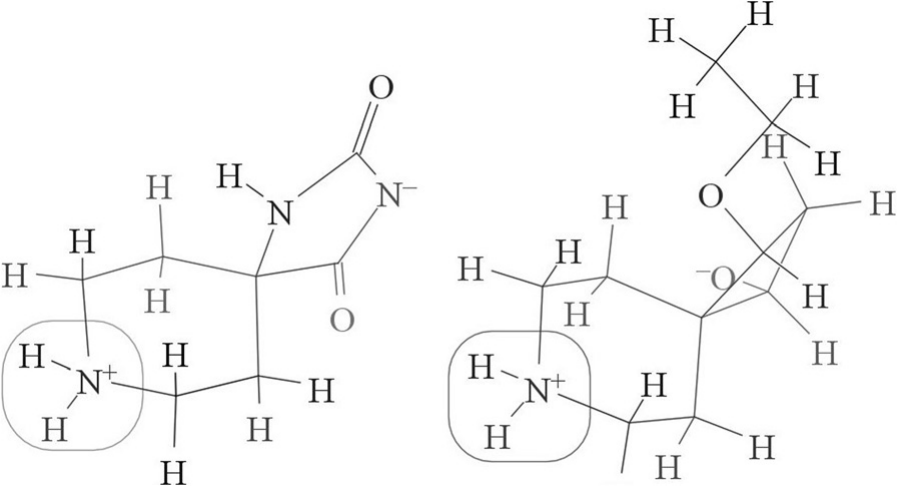
Examples of two bonds with identical descriptors in the final model, but very different BDEs due to remote group effects.

In Figure 5 we focus on two similar N-H bonds in two different molecules. The local environments are quite similar for the two bonds, and one might anticipate the bond dissociation energies to be similar. Surprisingly, they differ up to 28.80 kcal/mol at the B3LYP/6-311++G** level. Because the nearest difference starts 4 bonds away from the target N-H bonds, their BDEs should be controlled by a remote group effect. Because all the atoms and their connections are the same within 3 bonds, there will be no difference in the descriptors of the two bonds, and the ASNN predictions will be the same. Even if two bonds are only slightly different within 3 bonds, but the bond dissociation energies are controlled by remote group effects, the similar difficulties would appear. So we will not expect this model to predict BDEs controlled by remote group effects.

Finally, the new data-driven models are expected to be limited by the limitations of the DFT method that was used to generated the training set. For example, Feng et al. reported that B3LYP systematically underestimates BDEs [27] by about 1–4 kcal/mol – the models are therefore assumed to inherit the same systematic deviation.

## Conclusions

Knowledge could be automatically extracted from a data set of > 12,000 BDEs calculated with DFT methods by machine learning techniques such as Random Forests and Neural Networks. The models could be applied to an independent test set achieving a root mean square deviation of 5.29 kcal/mol, a mean absolute deviation of 3.35 kcal/mol, and *R*^2^ = 0.953. Similar quality of predictions was observed across different types of bonds (RMSD 1.94–7.58 kcal/mol) except for N-H, O-H, and N-N bonds. Predictions were particularly accurate for C-H bonds (RMSD 3.68 kcal/mol, MAD 2.20 kcal/mol). The deviations between the ASNN predictions and the DFT (B3LYP) values are quite close to reported deviations (for simpler molecules) between B3LYP calculations and experimental values (MAD 3–8 kcal/mol) [27,29]. Even for experimental BDEs, uncertainties of 1–2 kcal/mol are rather common. Differently from quantum methods, the new machine learning models can provide ultra-fast estimations of BDEs. The ASNN predictions also exhibited better accuracy than PM6, except for O-H, N-H, and N-N bonds. A reason for the more problematic prediction of these bonds may reside in their frequent involvement in hydrogen bonding – determined by 3D through-space interactions not currently encoded by our descriptors.

Comparison of B3LYP/6-311++G(d,p)//DFTB calculations with B3LYP/6-311++G(3df,2p)//B3LYP/6-31G(d,p) revealed a smaller deviation than the observed error for the ASNN predictions, which confirms that the chosen method for building the database of theoretical calculations is a reasonable compromise between accuracy and computational cost.

This work combines chemoinformatics and theoretical chemistry methodologies utilizing the currently available computational power. We believe it demonstrates a way to use high-level theoretical quantum calculations in large-scale applications that otherwise would not afford the intrinsic computational cost.

## Abbreviations

2D: 2-Dimensional; ASE: Atomic simulation environment; ASNN: Associative Neural Network; B3LYP: Becke 3-parameter Lee-Yang-Parr; BDE: Bond dissociation energy; BFGS: Broyden-flectcher-goldfarb-shanno; CCSD(T): Coupled cluster with single and double and perturbative triple excitations; CDK: Chemistry development kit; DFT: Density functional theory; DFTB: Density functional based tight-binding; FFNN: Feed-forward neural network; FIRE: Fast inertial relaxation engine; HF: Hartree-fock; LOO: Leave-one-out; MAD: Mean absolute deviation; NN: Neural network; OOB: Out-of-bag; QCISD(T): Quadratic configuration interaction with single and double and perturbative triple excitations; RF: Random forest; RMSD: Root mean square deviation; SCC-DFTB: Self-consistent-charge density functional tight-binding; ZPE: Zero point energy

## Competing interests

The authors declare that they have no competing interests.

## Authors’ contributions

XQ gathered the data, performed the quantum chemistry calculations, implemented the descriptors, trained the models and developed the web interface, DARSL participated in the development of machine learning models, and JAS planned and coordinated the work. All authors read and approved the final manuscript.

## Supplementary Material

Additional file 1 Molecular structures with the calculated BDEs used for training machine learning methods. Click here for file
